# Predictive value of *XPG* rs2296147T>C polymorphism on clinical outcomes of cancer patients

**DOI:** 10.18632/oncotarget.11664

**Published:** 2016-08-29

**Authors:** Yuhan Wang, Yingying Han, Qiang Weng, Zhengrong Yuan

**Affiliations:** ^1^ College of Biological Sciences and Technology, Beijing Forestry University, Beijing 100083, People's Republic of China

**Keywords:** XPG, rs2296147, cancer, clinical outcomes, meta-analysis

## Abstract

The Xeroderma pigmentosum complementation group G (*XPG*) rs2296147T>C polymorphism is suspected to associate with the clinical outcomes of cancer patients. However, the results are inconsistent. This meta-analysis aimed to evaluate the reliable predictive value of *XPG* rs2296147T>C polymorphism on clinical outcomes of cancer patients. A total of 11 eligible studies were enrolled in this meta-analysis. Our results indicated that the cancer patients with TT and CT genotypes were significantly associated with better respond rates when compared with the CC genotype (TT versus (vs.) CC: odds ratio (OR) = 2.05, 95% confidence intervals (CIs), 1.32-3.20, P = 0.002; TT+CT vs. CC: OR= 1.57, 95% CI, 1.14-2.17, P = 0.005). The TT genotype and/or T allele might be associated with higher survival time for cancer patients than the CC genotype and/or C allele. The cumulative meta-analyses showed an apparent beneficial objective response of TT genotype on cancer patients. In conclusion, this meta-analysis suggests that the *XPG* rs2296147T>C polymorphism is associated with the clinical outcomes of cancer patients. The *XPG* rs2296147T>C polymorphism might be a predictive factor of prognosis in cancers patients and contribute to individual treatment in the future.

## INTRODUCTION

Nowadays, cancer has emerged as one of the most serious public health problems worldwide [1–3]. Despite intensive efforts have been made to improve the efficacy of cancer diagnosis and therapy, the overall survival (OS) time of cancer patients is still short [1–3]. It is very necessary to identify more reliable biomarkers for early diagnosis, accurate prognosis prediction, and efficacy for cancer patients [2]. Emerging evidence has demonstrated that genetic factors are considered to influence the cancer development, treatment effectiveness, survival time of cancer patients, therefore affect the prognosis of patients [3, 4]. It has been speculated that DNA damage was significantly associated with the DNA repair capacity [5–7]. The genetic variants in DNA repair genes alter the activity of DNA repair, thus influence the effectiveness of therapy, prognosis and survival of patients [1, 3, 4, 6]. The DNA repair genes have been identified in different DNA repair pathways [5–7]. The nucleotide excision repair (NER) pathway is the most versatile repair mechanism responsible for repairing bulky DNA damage [5]. The xeroderma pigmentosum complementation group G (*XPG*), also known as excision repair cross-complementation group 5(*ERCC5*), is one of the critical DNA repair enzymes of NER pathway. *XPG* gene is located on chromosome 13q32-q33, and encodes a protein of 1186 amino acids, which containing 15 exons and 14 introns. Previous studies indicate that the XPG rs2296147T>C polymorphism is suspected to have relationship with the clinical outcomes of cancer patients, such as colorectal cancer (CRC) [8–11], epithelial ovarian cancer (EOC) [12, 13], head and neck cancer(HNC) [14], non-small cell lung cancer (NSCLC) [15–19], gastric cancer (GC) [20] and osteosarcoma (OC) [21, 22]. Published data from these studies have shown inconsistent results. However, a systematic review and meta-analysis is still lacking. Thus, the aim of this meta-analysis was designed to summarize the currently available published findings and comprehensively assess the reliable predictive value of *XPG* rs2296147T>C polymorphism on clinical outcomes of cancer patients.

## RESULTS

### Studies characteristics

**Figure 1:**
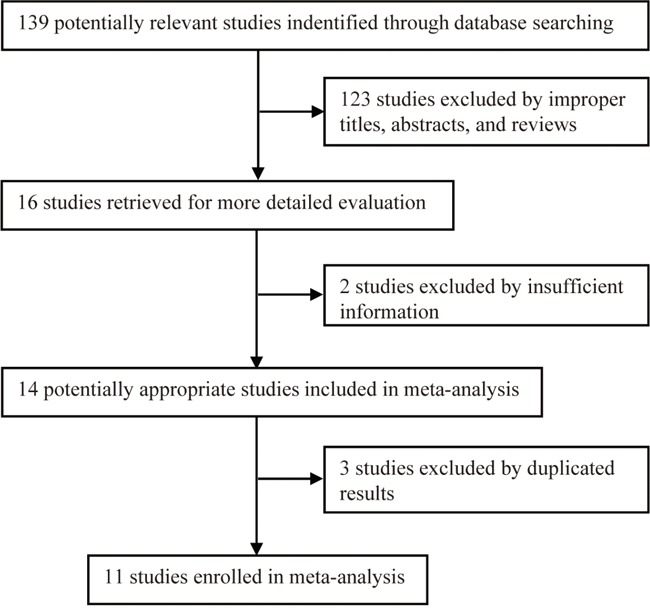
The flow diagram of the study selection process in meta-analysis

Our initial systematic search retrieved 139 publications using different combinations of key terms. Based on our inclusion and exclusion criteria, eleven eligible publications were ultimately enrolled for the data pool in this meta-analysis [8, 9, 11, 13–19, 22] (Figure 1), altogether 5316 cancer patients. The general characteristics of enrolled studies are summarized in Table 1. These studies included five NSCLC studies, three CRC studies, one EOC study, one HNC study, and one OC study (Table 1). Ten studies were conducted on Asian patients, and one was conducted on Caucasian patients (Table 1). Eight studies were published in English and three studies were published in Chinese. Of these studies, five studies reported the objective response rate (ORR), eight studies reported the OS and hazard ratios (HRs), and seven studies reported the median progression-free survival (PFS) and HRs (Table 2). The sample sizes of included studies ranged from 105 to 1901 cancer patients. Several genotyping methods were used in the enrolled studies, including the polymerase chain reaction (PCR)-ligase detection reaction (PCR-LDR), PCR-restriction fragment length polymorphism (PCR-RFLP), Taqman real-time-PCR (Taqman RT-PCR), and Illumina GoldenGate assay with Sentrix Array Matrix and 96-well standard microtiter plates (Table 1).

**Table 1:** The characteristics of enrolled studies in the meta-analysis

Study	Year	Country	Ethnicity	Cancer type	Number of patients	Median age (year)	Clinical stage	Evaluation criterion	Clinical outcomes	Genotyping methods	Genotype distribution		
											TT	CT	CC
**Chen et al.,**	2009	China	Asian	CRC	105	55 (29−84)	Advanced	RECIST	ORR/TTP	PCR-LDR	27/42^a^	12/18^a^	2/4^a^
**Chen et al.,**	2011	China	Asian	CRC	105	55 (29−84)	Advanced	RECIST	PFS	PCR-LDR	-	-	-
**He et al.,**	2013	China	Asian	NSCLC	228	60 (19−84)	III-IV	WHO	ORR	PCR-RFLP	61/85^a^	28/44^a^	2/7^a^
**Bai et al.,**	2013	China	Asian	OC	185	16.8 (6−39)	NR	NR	ORR/OS	Sequenome MassARRAY platform	29/8^a^	31/18^a^	46/53^a^
**Zhang et al.,**	2013	China	Asian	NSCLC	475	64.3 (31.7−76.1)	IIIA/B-IV	European Organization for Research and Treatment of Cancer	ORR/OS/PFS	Taqman RT-PCR	21/37^a^	45/99^a^	71/178^a^
**Yi et al.,**	2013	China	Asian	NSCLC	433	61.4 (32.5−78.7)	IIIA/B-IV	NR	OS/PFS	Taqman RT-PCR	-	-	-
**Hu et al.,**	2014	China	Asian	NSCLC	277	63.1 (28.7−74.5)	IIIA/B-IV	WHO	OS/PFS	PCR-RFLP	-	-	-
**Wyss et al.,**	2014	USA	Caucasian	HNC	1227	NR	I-IV	NR	OS/OD/DSS	Illumina GoldenGate assay with Sentrix Array Matrix and 96-well standard microtiter plates	-	-	-
**Zou et al.,**	2015	China	Asian	NSCLC	246	64.3 (31.7−76.1)	IIIA/B-IV	WHO	OS/PFS	PCR-RFLP	-	-	-
**Hu et al.,**	2015	China	Asian	EOC	239	NR	I-IV	NR	ORR/OS/PFS	PCR-LDR	90/51^a^	69/29^a^,^b^	-
**Wang et al.,**	2016	China	Asian	CRC	1901	57.1 (13−91)	NR	NR	OS/PFS	Taqman RT-PCR	-	-	-

Abbreviations: CRC, colorectal cancer; DDS, disease-specific survival; EOC, epithelial ovarian cancer; HNC, Head and neck cancer; MST, median survival time; NR, not reported; NSCLC, non-small cell lung cancer; OC, osteosarcoma; OD, overall deaths; ORR, objective response rate; OS, overall survival; PCR-LDR, polymerase chain reaction (PCR)-ligase detection reaction; PCR-RFLP, PCR-restriction fragment length polymorphism; PFS, progression-free survival; RECIST, Response Evaluation Criteria in Solid Tumors; Taqman RT-PCR, Taqman real-time-PCR; TTP, time to progression (months); WHO, World Health Organization.

a Number of patients for ORR; in front of oblique line is good responder (complete response (CR) + partial response (PR)) and behind oblique line is poor responder (stable disease (SD) + progressive disease (PD)).

b Number of patients for CT and TT genotypes.

**Table 2:** Association between *XPG* rs2296147T>C polymorphism and overall survival and progression-free survival

Study	Year	HR	TT	CT	CC	TT+CT	CT+CC	T
**Chen et al.,**	2011	OS	-	-	-	-	-	-
		PFS	1.23(0.73−2.07)	-	-	-	1(Reference)	-
**Bai et al.,**	2013	OS	0.47(0.17−0.94)	1.36(0.64−2.85)	1(Reference)	0.84(0.58−1.55)	-	-
		PFS	-	-	-	-	-	-
**Zhang et al.,**	2013	OS	0.50(0.27−0.95)	0.72(0.46−1.16)	1(Reference)	-	-	-
		PFS	0.75(0.38−1.55)	0.90(0.55−1.50)	1(Reference)	-	-	-
**Yi et al.,**	2013	OS	-	-	1(Reference)	0.66(0.48−0.99)	-	-
		PFS	-	-	1(Reference)	0.73(0.51−0.97)	-	-
**Hu et al.,**	2014	OS	0.47(0.22−0.93)	0.79(0.51−1.23)	1(Reference)	-	-	0.49(0.36-0.68)^a^
		PFS	0.48(0.24−0.93)	0.85(0.56−1.29)	1(Reference)	-	-	0.52(0.38-0.70)^a^
**Wyss et al.,**	2014	OS	1(Reference)	-	-	-	0.78(0.62−0.97)^b^	-
		PFS	-	-	-	-	-	-
		OS	1(Reference)	-	-	-	1.07(0.77−1.48)^c^	-
		PFS	-	-	-	-	-	
**Zou et al.,**	2015	OS	0.32(0.14−0.71)	0.89(0.46−1.76)	1(Reference)	-	-	0.54(0.32−0.98)^a^
		PFS	0.31(0.13−0.73)	0.55(0.27−1.12)	1(Reference)	-	-	0.44(0.24−0.83)^a^
**Hu et al.,**	2015	OS	1(Reference)	-	-	-	0.50(0.28−0.87)	-
		PFS	1(Reference)	-	-	-	0.63(0.41−0.98)	-
**Wang et al.,**	2016	OS	1.49(0.92−2.43)	1.38(0.84−2.28)	1(Reference)	1.21(0.95−1.54)	-	-
		PFS	1.74(1.08−2.80)	1.78(1.09−2.90)	1(Reference)	1.32(1.05−1.67)	-	-

Abbreviations: HR: hazard ratio; OS, overall survival; PFS, progression-free survival.

a HR for T versus C.

b HR for Whites.

c HR for African Americans.

### Objective response of *XPG* rs2296147T>C polymorphism

**Figure 2:**
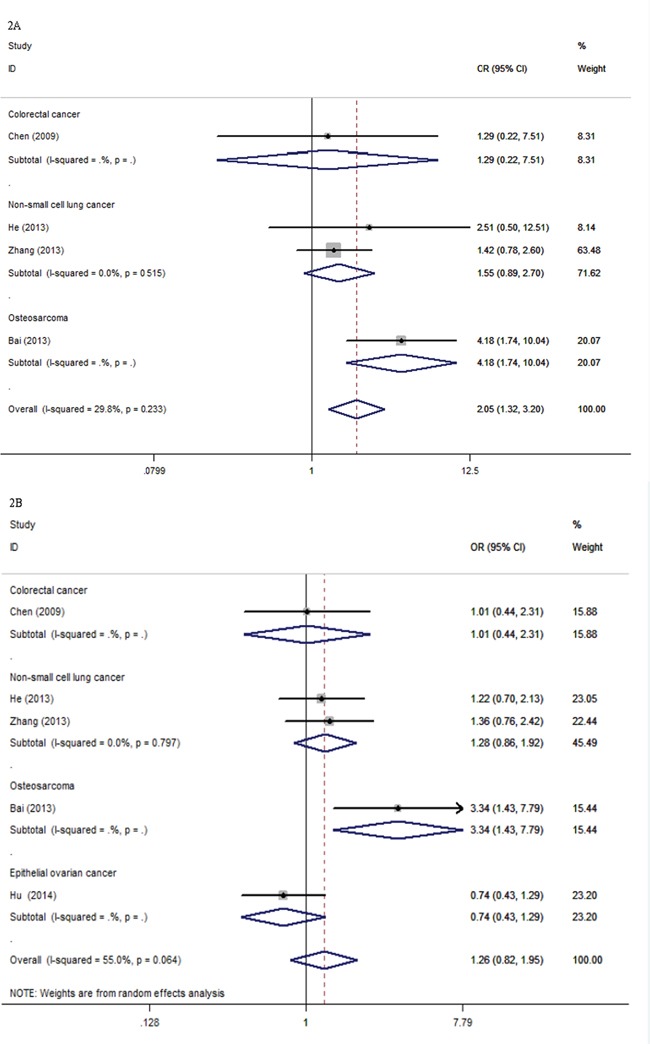
Forest plots for the association between *XPG* rs2296147T>C polymorphism and objective response rate of cancer patients CI: confidence interval; ORR: objective response rate; ORs: odds ratios; vs.: versus. Figure 2A. ORR (ORs and 95% CI) under TT vs. CC in cancer type. Figure 2B. ORR (ORs and 95% CI) under TT vs. CT+CC in cancer type.

A total of five eligible studies, including 1232 cancer patients, were qualified for the final analysis for objective response of *XPG* rs2296147T>C polymorphism. We observed significant associations between objective response and *XPG* rs2296147T>C polymorphism (TT versus (vs.) CC: odds ratio (OR) = 2.05, 95% confidence intervals (CIs), 1.32-3.20, P = 0.002, Figure 2A; TT+CT vs. CC: OR = 1.57, 95% CI, 1.14-2.17, P = 0.005, Table 3). However, there were no significant associations under other genetic models (CT vs. CC: OR = 1.38, 95% CI, 0.97-1.97, P = 0.078; TT vs. CT+CC: OR = 1.26, 95% CI, 0.82-1.95, P = 0.302, Figure 2B; T vs. C: OR= 1.46, 95% CI, 0.99-2.17, P = 0.058, Table 3). In the stratified analyses by cancer type, an evidently increased risk was found in the OC (TT vs. CC: OR = 4.18, 95% CIs, 1.74-10.04, P = 0.001, Figure 2A; TT+CT vs. CC: OR = 2.66, 95% CI, 1.45-4.88, P = 0.002; TT vs. CT+CC: OR = 3.34, 95% CI, 1.43-7.79, P = 0.005, Figure 2B; T vs. C: OR= 2.64, 95% CI, 1.65-4.21, P < 0.001, Table 3). The cancer patients bearing the favorable T allele, TT and CT genotypes were associated with better respond rates compared to those with the unfavorable C allele and CC genotype (Table 3).

**Table 3:** Meta-analysis of the association between *XPG* rs2296147T>C polymorphism and objective response rate, overall survival and progression-free survival

Genetic comparisons	Study groups	No. of studies^a^	Test of association				Test of Heterogeneity		
			OR^b^/HR^c^ (95% CI)	Z	P-value	Model	χ^2^	P-value	I^2^(%)
Objective response rate									
TT vs. CC	Overall	4	2.05(1.32−3.20)	3.17	0.002	F	4.28	0.233	29.8
	Cancer type								
	CRC	1	1.29(0.22−7.51)	0.28	0.780	F	-	-	-
	NSCLC	2	1.55(0.89−2.70)	1.53	0.125	F	0.42	0.515	0
	OC	1	4.18(1.74−10.04)	3.20	0.001	F	-	-	-
CT vs. CC	Overall	4	1.38(0.97−1.97)	1.76	0.078	F	2.06	0.560	0
	Cancer type								
	CRC	1	1.33(0.21−8.46)	0.31	0.760	F	-	-	-
	NSCLC	2	1.20(0.78−1.85)	0.84	0.400	F	0.60	0.440	0
	OC	1	1.98(0.98−4.01)	1.91	0.056	F	-	-	-
TT+CT vs. CC	Overall	4	1.57(1.14−2.17)	2.79	0.005	F	4.77	0.190	37.0
	Cancer type								
	CRC	1	1.30(0.23−7.44)	0.29	0.768	F	-	-	-
	NSCLC	2	1.28(0.87−1.88)	1.23	0.217	F	0.67	0.413	0
	OC	1	2.66(1.45−4.88)	3.16	0.002	F	-	-	-
TT vs. CT+CC	Overall	5	1.26(0.82−1.95)	1.05	0.295	R	8.88	0.064	55.0
	Cancer type								
	CRC	1	1.01(0.44−2.31)	0.02	0.981	R	-	-	-
	NSCLC	2	1.28(0.86−1.92)	1.22	0.223	R	0.07	0.797	0
	OC	1	3.34(1.43−7.79)	2.79	0.005	R	-	-	-
	EOC	1	0.74(0.43−1.29)	1.06	0.290	R	-	-	-
T vs. C	Overall	4	1.46(0.99−2.17)	1.89	0.058	R	8.57	0.036	65.0
	Cancer type								
	CRC	1	1.05(0.53−2.11)	0.14	0.888	R	-	-	-
	NSCLC	2	1.24(0.95−1.60)	1.61	0.108	R	0.02	0.897	0
	OC	1	2.64(1.65−4.21)	4.07	< 0.001	R	-	-	-
**Overall survival**									
TT vs. CC	Overall	5	0.58(0.32−1.07)	1.75	0.080	R	16.00	0.003	75.00
	Cancer type								
	OC	1	0.47(0.20−1.11)	1.73	0.084	R	-	-	-
	NSCLC	3	0.44(0.29−0.66)	3.96	< 0.001	R	0.78	0.677	0
	CRC	1	1.49(0.92 −2.43)	1.61	0.106	R	-	-	-
CT vs. CC	Overall	5	0.94(0.74−1.19)	0.54	0.588	F	5.11	0.276	21.80
	Cancer type								
	OC	1	1.36(0.64−2.87)	0.81	0.420	F	-	-	-
	NSCLC	3	0.78(0.58−1.04)	1.70	0.089	F	0.27	0.875	0
	CRC	1	1.38(0.84−2.27)	1.26	0.206	F	-	-	-
TT+CT vs. CC	Overall	3	0.89(0.60−1.34)	0.54	0.540	R	7.89	0.019	74.60
	Cancer type								
	OC	1	0.84(0.51−1.37)	0.70	0.487	R	-	-	-
	NSCLC	1	0.66(0.46−0.95)	2.25	0.024	R	-	-	-
	CRC	1	1.21(0.95−1.54)	1.55	0.122	R	-	-	-
T vs. C	Overall	2	0.50(0.38−0.66)	4.89	< 0.001	F	0.09	0.767	0
CC+CT vs. TT	Overall	3	0.79(0.57−1.11)	1.34	0.180	R	5.67	0.059	64.70
	Cancer type								
	HNC	2	0.89(0.66−1.21)	0.73	0.467	R	2.45	0.118	59.20
	EOC	1	0.50(0.28−0.88)	2.40	0.017	R	-	-	-
**Progression-free survival**									
TT vs. CC	Overall	4	0.69(0.32−1.52)	0.91	0.361	R	16.76	0.001	82.10
	Cancer type								
	NSCLC	3	0.50(0.31−0.81)	2.84	0.005	R	2.47	0.291	18.90
	CRC	1	1.74(1.08−2.82)	2.28	0.023	R	-	-	-
CT vs. CC	Overall	4	0.96(0.62−1.50)	0.17	0.866	R	8.80	0.032	65.90
	Cancer type								
	NSCLC	3	0.81(0.60−1.08)	1.45	0.146	R	1.36	0.507	0
	CRC	1	1.78(1.09−2.90)	2.31	0.021	R	-	-	-
TT+CT vs. CC	Overall	2	0.99(0.56−1.77)	0.03	0.979	R	8.58	0.003	88.30
	Cancer type								
	NSCLC	1	0.73(0.53−1.01)	1.92	0.055	R	-	-	-
	CRC	1	1.32(1.05−1.67)	2.35	0.019	R	-	-	-
T vs. C	Overall	2	0.50(0.38-0.66)	4.91	< 0.001	F	0.22	0.636	0
CC+CT vs. TT	Overall	2	0.70(0.50−0.98)	2.10	0.035	F	0.53	0.468	0
	Cancer type								
	CRC	1	0.81(0.48−1.36)	0.79	0.428	F	-	-	-
	EOC	1	0.63(0.41−0.97)	2.08	0.038	F	-	-	-

Note: CI, confidence interval; CRC, colorectal cancer; EOC, epithelial ovarian cancer; HNC, Head and neck cancer; HR, hazard ratio; NSCLC, non-small cell lung cancer; OC, osteosarcoma; OR, odds ratio; vs., versus; F, fixed effect model; R, random effect model; Random effect model was chosen when P-value < 0.10 and/or I^2^ > 50% for heterogeneity test; otherwise fixed effect model was used.

a The detailed references are given in Table 1 and 2.

b The OR for objective response rate.

c The HR for overall survival and progression-free survival.

### Overall survival of *XPG* rs2296147T>C polymorphism

**Figure 3:**
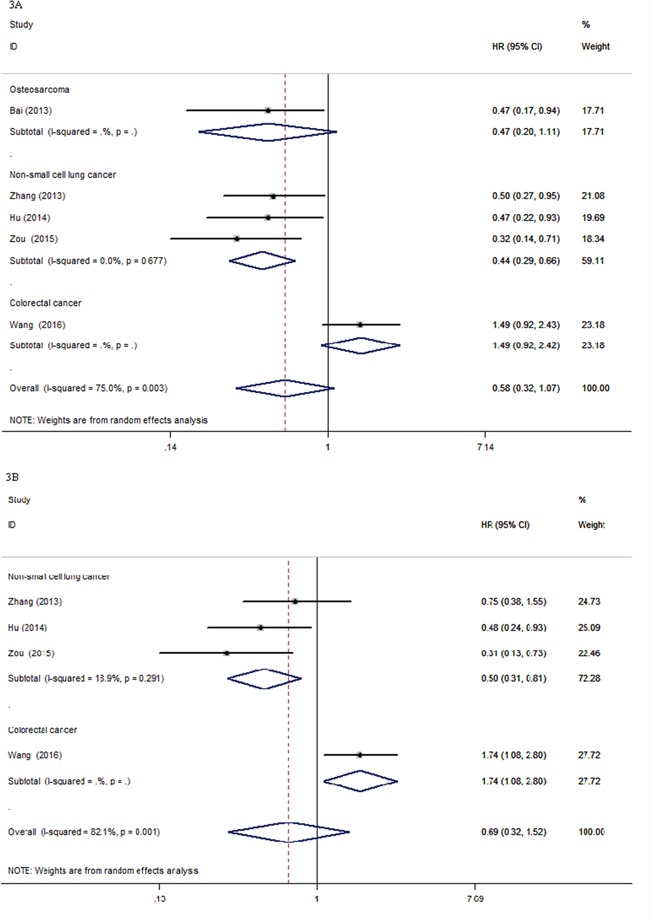
Forest plots for the association between *XPG* rs2296147T>C polymorphism and overall survival and progression-free survival of cancer patients CI: confidence interval; HRs: Hazard ratios; OS: overall survival; PFS: progression-free survival; vs.: versus. Figure 3A. OS (HRs and 95% CI) under TT vs. CC in cancer type. Figure 3B. PFS (HRs and 95% CI) under TT vs. CC in cancer type.

There were eight eligible studies with 4983 cancer patients, qualified for the final analysis of the relationship between the OS of cancer patients and *XPG* rs2296147T>C polymorphism. We detected a significant association between the OS of cancer patients and *XPG* rs2296147T>C polymorphism under T vs. C genetic model (HR = 0.50, 95% CI, 0.38-0.66, P < 0.001, Table 3). The stratified analyses by cancer type showed that the *XPG* rs2296147T>C polymorphism was statistically significantly associated with the OS of cancer patients in NSCLC (TT vs. CC, HR = 0.44, 95% CI, 0.29-0.66, P < 0.001, Figure 3A; TT+CT vs. CC: HR = 0.66, 95% CI, 0.46-0.95, P = 0.024, Table 3). We also observed a significant association between the OS of EOC and *XPG* rs2296147T>C polymorphism under CC+CT vs. TT genetic model (HR = 0.50, 95% CI, 0.28-0.88, P = 0.017, Table 3). Our results indicated that the TT genotype and/or T allele might be associated with higher survival time for cancer patients than the CC genotype and/or C allele (Table 3).

### Progression-free survival of *XPG* rs2296147T>C polymorphism

Seven studies with a total number of 3676 cancer patients were recruited for the final analysis of the relationship between the PFS of cancer patients and *XPG* rs2296147T>C polymorphism. The *XPG* rs2296147T>C polymorphism was significantly associated with the PFS of cancer patients (CC+CT vs. TT, HR = 0.70, 95% CI, 0.50-0.98, P = 0.035; T vs. C, HR = 0.50, 95% CI, 0.38-0.66, P <0.001, Table 3). When stratified by cancer type, a significant association between the *XPG* rs2296147T>C polymorphism and PFS of NSCLC patients was found under TT vs. CC genetic model (HR = 0.50, 95% CI, 0.31-0.81, P = 0.005, Figure 3B, Table 3). There was a significant association between the *XPG* rs2296147T>C polymorphism and PFS of EOC patients under CC+CT vs. TT genetic model (HR = 0.63, 95% CI, 0.41-0.97, P = 0.038, Table 3). We also detected significant associations between the *XPG* rs2296147T>C polymorphism and PFS of CRC patients (TT vs. CC, HR = 1.74, 95% CI, 1.08-2.82, P = 0.023, Figure 3B; CT vs. CC, HR = 1.78, 95% CI, 1.09-2.90, P = 0.021; TT+CT vs. CC, HR = 1.32, 95% CI, 1.05-1.67, P = 0.019, Table 3).

No other significant associations between the *XPG* rs2296147T>C polymorphism and clinical outcomes of cancer patients were observed in this meta-analysis (Table 3).

### Publication bias and sensitivity analysis

No obvious publication bias was found through either the inverted funnel plots or Begg's test (data not shown). In the sensitivity analysis, changing the effect models had no significant effects on the pooled ORs/HRs, and did not influence the final strength of association between XPG rs2296147T>C polymorphism and clinical outcomes of cancer patients. The influence of excluding each study on the pooled ORs/HRs was recalculated by repeating the meta-analysis, and the results indicated that excluding each study did not significantly change the overall effects, suggesting that our results are credible.

### Cumulative meta-analyses

**Figure 4:**
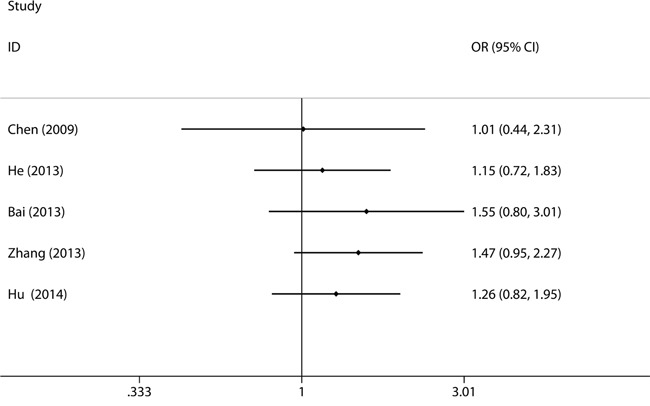
Forest plot for cumulative meta-analysis to sort out the time-tendency of clinical outcomes in cancer patients for *XPG* rs2296147T>C polymorphism CI: confidence interval; ORR: objective response rate; ORs: odds ratios; vs.: versus. ORR (ORs and 95% CI) under TT vs. CT+CC.

The cumulative meta-analyses based on year of publication showed an apparent beneficial objective response of TT genotype on cancer patients in the recent studies. As shown in Figure 4, between 2009 and 2014, five studies were enrolled, resulting in an overall effect estimate of OR being 1.26 (95% CI, 0.82-1.95, TT vs. CT+CC, Figure 4).

## DISCUSSION

The NER is one of the major pathways of the DNA repair system, and the *XPG* gene is an indispensable component of the NER system. Many of SNPs in *XPG* gene have been reported to be associated with the outcomes of various cancers [5, 8–29]. The association between XPG rs2296147T>C polymorphism and clinical outcomes of cancer patients has been widely studied [8, 9, 11, 13–19, 22]. Bai et al observed that OC patients with XPG rs2296147T>C TT genotype were associated with better response to chemotherapy and longer OS than CC genotype (OR (95% CI) for ORR: 4.17 (1.64-11.54); HR (95% CI) for OS: 0.47 (0.17-0.94)) [22]. The *XPG* polymorphisms could be used as predictive markers for prognosis of OC [22]. Zhang et al found a significant decreased risk of death from NSCLC among patients carrying the XPG rs2296147T>C TT genotype when compared with the CC genotype (HR (95% CI) for OS: 0.50 (0.27-0.95)) [18]. The XPG rs2296147T>C polymorphism is associated with response to platinum-based chemotherapy in advanced NSCLC, and could be predictive of prognosis [18]. Yi et al indicated that NSCLC patients carrying XPG rs2296147T>C TT+CT genotype had a significantly longer median PFS and OS than CC genotype (HR (95% CI) for PFS: 0.73(0.51-0.97); HR (95% CI) for OS: 0.66(0.48-0.99)). The XPG rs2296147 T>C TT+CT genotype contribute to the better survival of NSCLC [19]. Hu et al reported that individuals carrying XPG rs2296147T>C T allele was associated with better PFS and OS of NSCLC (HR (95% CI) for PFS: 0.52 (0.38-0.70); HR (95% CI) for OS: 0.49(0.36-0.68)) [16]. Zou et al indicated that patients carrying the XPG rs2296147T>C TT genotype had a significantly reduced risk of developing progressive disease or dying from NSCLC (HR (95% CI) for PFS: 0.31 (0.13-0.73); HR (95% CI) for OS: 0.32 (0.14-0.71)) [15]. These studies suggest that the XPG rs2296147T>C polymorphism could be used as surrogate markers toward individualizing NSCLC treatment strategies [15, 16, 19]. However, He et al reported that there was no statistically significant association between XPG rs2296147T>C polymorphism and treatment response in NSCLC [17]. In addition, Chen et al suggested that no significant association was found between the XPG rs2296147T>C polymorphism and chemotherapy response in patients with advanced CRC [9, 10]. Hu et al observed that the XPG rs2296147T>C C allele was associated with the PFS and OS of EOC compared with the TT genotype (HR (95% CI) for PFS: 0.63 (0.41~0.98); HR (95% CI) for OS: 0.50 (0.28~0.87)) [12]. The XPG rs2296147T>C polymorphism may play as the marker in predicting the clinical outcomes of EOC treated with platinum chemotherapy [12]. Wang reported that CRC patients carrying the *XPG* rs2296147T>C TT+CT genotype had a significantly shorter median 10 years PFS than those carrying CC genotype (HR = 1.324, 95% CI, 1.05–1.67) [8]. The XPG rs2296147T>C polymorphism could be predictive of unfavorable prognosis of CRC patients [8]. Results from these published studies remain conflicting rather than conclusive.

In the present study, we firstly conducted a systematic meta-analysis to assess the predictive value of XPG rs2296147T>C polymorphism on clinical outcomes of cancer patients. Results from this study could provide more reliable and precise comprehensive evaluation. Fortunately, we are delighted to find that the cancer patients carrying XPG rs2296147T>C T allele are associated with higher survival time and lower risks of death compared with those carrying C allele. Our cumulative meta-analysis indicated a distinct trend toward an apparent better respond rate for TT genotype of XPG rs2296147T>C polymorphism in cancer patients, which also showed the stable time-dependent trend (Figure 4). The sequential year-to-year cumulative meta-analyses could have made the evidence much clearer earlier. It may help planning further clinical trials, preventing many redundant trials, redirecting trial design, and leading to sufficient assessment of clinical outcomes. Thus, findings from this cumulative meta-analysis about the response rates of *XPG* rs2296147T>C polymorphism in cancer patients should be weighed with caution. Our findings suggested that the *XPG* rs2296147T>C polymorphism could be a predictive factor for clinical outcomes of human cancers. The *XPG* rs2296147T>C polymorphism might be used as a biomarker in personalized cancer treatment strategies.

Some potential limitations in this meta-analysis should be noted when interpreting our findings. Firstly, although the sample size of our study was relatively large by collecting all enrolled studies, the statistical power was still limited in the stratified analyses because of the relatively small sample sizes. Some of the findings in stratified analyses may have been undervalued, because only one trial was available for analyses. Many important confounding factors were not taken into account in the stratified analyses, such as age, gender, ethnicities, cancer pathology types, cancer stage, cancer history, smoking habit, specific anti-cancer drugs, treatment regimens and test methods. These factors could contribute to the clinical outcomes of cancer patients. However, few of included studies provided sufficient data for stratified analyses, thus making such stratified analyses impossible. Secondly, some significant heterogeneity between studies did observed. However, this heterogeneity was not significantly change the main conclusions, because our findings reflected the most current state of this issue in published studies. Our analyses were based on unadjusted estimates, since not all published studies calculating adjusted estimates. Thirdly, all of included studies were performed in Asians (Chinese), excepting one study in Caucasian populations (Whites and African Americans). Fourthly, only published studies in Chinese and English were included, published studies in other languages, ongoing studies and unpublished data were not obtained. These may have caused some biases in our estimates. Therefore, to make the result more accurate and reliable, further studies should avoid these pitfalls.

## CONCLUSIONS

In conclusion, despite the limitations, this meta-analysis indicates that the *XPG* rs2296147T>C polymorphism is associated with the clinical outcomes of cancer patients. Our findings suggest a predictive role for *XPG* rs2296147T>C polymorphism in clinical outcomes of cancer patients. The *XPG* rs2296147T>C polymorphism should be considered as a prognostic factor in human cancers. Further well-designed functional studies with large sample sizes in diverse ethnic populations will be necessary to validate these findings in the future.

## MATERIALS AND METHODS

### Literature search strategies

We systematically retrieved the relevant literatures using the following search terms “*XPG* or Xeroderma pigmentosum complementation group G”, “ERCC5 or Excision repair cross-complementation group 5”, “rs2296147”, ‘‘SNP or genetic polymorphism or variation”, and “cancer or tumour or tumor or neoplasm or carcinoma” from the PubMed, ISI Web of Science, Excerpta Medica Database (EMBASE), Google Scholar, Wiley Online Library, ScienceDirect, Springer, VIP Database for Chinese Technical Periodicals, WANFANG Data, and Chinese National Knowledge Infrastructure (CNKI) databases (the last search was updated on May 20, 2016). We searched the articles without the restriction of country, race, languages and publication date. Reference lists were manually searched for further additionally relevant studies.

### Inclusion and exclusion criteria

The inclusion criteria were as follows: (1). All patients diagnosed with cancer should be histologically confirmed; (2). *XPG* rs2296147T>C polymorphism should be genotyped; (3). Treatments and clinical outcomes (i.e., ORR, OS, PFS) should be reported; (4). Sufficient data should be provided to estimate relative risks (i.e., ORs and HRs with corresponding to 95% CIs) for prognostic effects of cancer patients; (5). Only full-text articles were enrolled. The exclusion criteria were utilized to exclude the literatures: (1). Abstracts, letters, comments, reviews and meta-analysis; (2). Duplicated studies; (3). Not reported sufficient data; (4). The corresponding authors were not provided the relevant information upon our request.

### Data extraction

Two investigators (Yuhan Wang and Zhengrong Yuan) independently extracted data from the included studies. The discrepancies between investigators were discussed and solved with consensus from our team's decision. The following information was extracted: the first author's name, year of publication, country, ethnicities, cancer type, number of patients, median age (year), clinical stage, evaluation criterion, clinical outcomes (ORR, OS, PFS, time to progression (TTP), overall deaths (OD), disease-specific survival (DSS), HRs with corresponding to 95% CIs), genotyping methods, and the number of responders and non-responders in different genotypes.

### Statistical analysis

We performed this meta-analysis according to the PRISMA guidelines (Figure S1 and Table S1) [30]. The ORR, OS, PFS, ORs, HRs with corresponding to 95% CIs were extracted from the included articles. The ORs and 95% CIs were calculated for the objective response vs. no response after treatment (complete response (CR) + partial response (PR) vs. progressive disease (PD) + stable disease (SD)). The HRs with corresponding to 95% CIs of OS and PFS were evaluated from the raw data of enrolled studies. We evaluated the association between *XPG* rs2296147T>C polymorphism and clinical outcomes of cancer patients by pooled ORs/HRs with corresponding to 95% CIs under different genetic models (TT vs. CC, CT vs. CC, TT+CT vs. CC, TT vs. CT+CC, T vs. C, CC+CT vs. TT). Then, we conducted the stratification analyses by cancer type. The significance of pooled ORs/HRs was assessed by the Z-test. The between-study heterogeneity was assessed with the Cochran's chi-square-based Q-test [31, 32] and I^2^ index [33]. The P-value > 0.10 and/or I^2^ index < 50% indicates no significant heterogeneity between studies [34], so the pooled ORs/HRs were evaluated using the fixed-effects model (the Mantel-Haenszel method) [35]. Otherwise, the random-effects model with the DerSimonian and Laird method was utilized [36]. The potential publication bias was investigated by the inverted funnel plots and Begg's test [37]. The sensitivity analysis was examined by changing the effect models and excluding each study to recalculate the ORs/HRs and 95% CIs. To sort out the time-tendency of clinical outcomes of cancer patients, we conducted a sequential year-to-year cumulative meta-analyses. All statistical analyses were carried out with the STATA software version 11.0 (STATA Corporation, College Station, TX, USA). All P-values were two-sided test with a significant level of P < 0.05.

## SUPPLEMENTARY MATERIALS FIGURE AND TABLE




