# Supplementary material for: Predictive value of XPG rs2296147T>C polymorphism on clinical outcomes of cancer patients
Source: Oncotarget. 2016 Aug 29;7(40):65770–81. doi: 10.18632/oncotarget.11664 (PMC5323191; doi:10.18632/oncotarget.11664)
Supplement: Supplementary file 1 [file oncotarget-07-65770-s001.pdf]

Predictive value of *XPG* rs2296147T>C polymorphism on clinical outcomes of cancer patients

SUPPLEMENTARY FIGURE AND TABLE

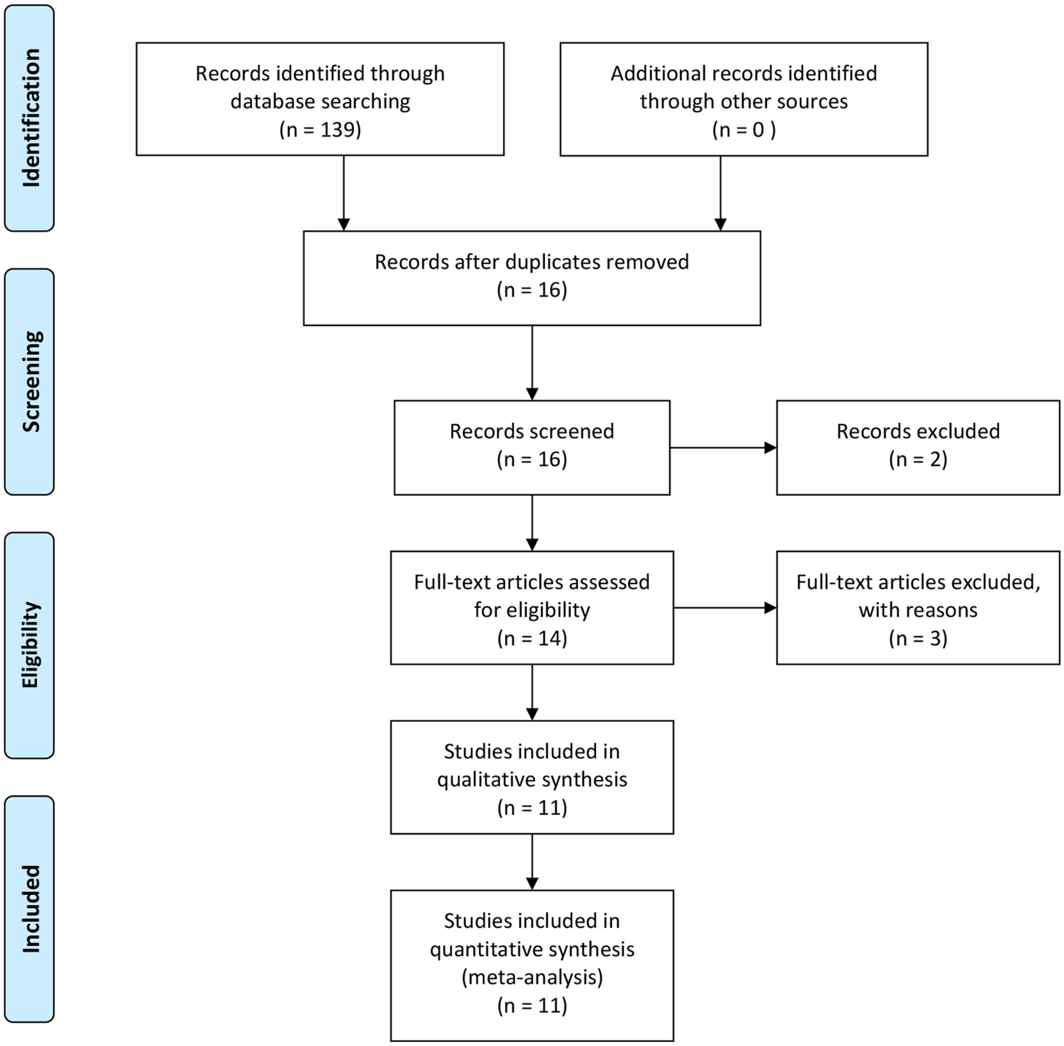

Supplementary Figure S1: PRISMA 2009 Flow Diagram.

Supplementary Table S1: PRISMA 2009 Checklist (DOC)

See Supplementary File 1
